# INCIDENCE OF COGNITIVE IMPAIRMENT AMONG U.S. CHINESE OLDER ADULTS: DOES SOCIAL ENGAGEMENT MATTER?

**DOI:** 10.1093/geroni/igz038.3381

**Published:** 2019-11-08

**Authors:** Dexia Kong, XinQi Dong

**Affiliations:** Rutgers University, New Brunswick, New Jersey, United States

## Abstract

The increasing diversity in U.S. aging population warrants improved understanding of risk factors of cognitive aging in minority populations. This study presents the prevalence of incident cognitive impairment (CI) among U.S. Chinese older adults; and the relationship between social engagement and incident CI. Data were obtained from the Population-based Study of Chinese Elderly in Chicago, a prospective cohort study of Chinese older adults. Baseline (collected between 2011 and 2013) and one subsequent wave of data (collected between 2013 and 2015) were used in analyses (N=2,713). Social engagement was measured by the frequency of participation in social and cognitive activities (range=0-65). Cognitive function was assessed by a battery of 5 validated instruments. Incidence of CI was defined as having a follow-up cognition score lower than 1.5 standard deviations below the mean baseline cognition score. Logistic regression analyses were conducted. Nearly 6% of the sample reported incident CI. Chinese older adults who are more socially-engaged had a lower likelihood of developing CI (odds ratio [OR] 0.94, 0.92-0.96). The relationship was consistent across cognitive domains, including episodic memory (OR 0.95, 0.92-0.97), working memory (OR 0.92, 0.88-0.95), and perceptual speed (OR 0.95, 0.92-0.98). Furthermore, older age (OR 1.12, 1.09-1.15), and lower education (OR 0.91, 0.87-0.96) were associated with incident CI. No significant association was observed between gender, income, marital status, household size, acculturation, medical morbidities, depressive symptoms, and incident CI. The findings highlight the importance of social engagement in cognitive aging. Discrepancies with prior literature and implications of these findings will be discussed.

